# Effect of *Foeniculum Vulgare* Aqueous and Alcoholic Seed Extract against Zoonotic Cutaneous Leishmaniasis

**DOI:** 10.4314/ejhs.v31i2.23

**Published:** 2021-03

**Authors:** Gholamrezaei Mostafa, Jalallou Nahid, Seyyedtabaei Seyyed javad, Dadashi Alireza, Salimi Sabour Ebrahim

**Affiliations:** 1 Department of Medical Laboratory Sciences, AJA University of Medical Sciences, Tehran, Iran; 2 Department of Parasitology and Mycology, School of Medicine, Shahid Beheshti University of Medical Sciences, Tehran, Iran; 3 Department of Infectious Diseases, School of Medicine, AJA University of Medical Sciences, Tehran, Iran; 4 Department of Pharmacognosy, School of Pharmacy, Shahid Beheshti medical sciences, Tehran, Iran

**Keywords:** cutaneous leishmaniasis, Foeniculum vulgare seed extract, Leshimania, Major

## Abstract

**Background:**

Cutaneous leishmaniasis is considered one of the major neglected tropical diseases. Drug resistance, limitary efficacy, and severe side effects remain a challenge for treatment. Foeniculum vulgare is known as a medicinal plant belonging to the Apiaceae, and anti-microbial properties of this plant have already been confirmed.

**Method:**

The F.vulgare sterile aqueous and alcoholic extracts were prepared. In vitro has used RAW 264.7 cell line and L. major parasite (MRHO/IR/75/ER). Cytotoxicity assay on macrophages (CC50), cytotoxicity assay on promastigotes (IC50), and cytotoxicity assay on infected macrophages (EC50) were accomplished with both extracts by MTT and light microscopy methods. Four in vivo were allocated in four groups and five BALB/c mice each group. Stationary phase promastigotes were inoculated into the base of mice tails subcutaneously (SC). Measurement of the body weight, lesion size, parasite burden of the lesion, and spleen after 4 weeks for evaluation effects of the alcoholic extract on CL was done.

**Results:**

The results of in vitro revealed that the optimal concentrations of both extracts reducing the promastigotes and amastigotes growth. Alcoholic extract no harmful side effects for the host macrophages, while were indicated has a potent action against L. major. In vivo results after 4 weeks did not show any variation in lesion size and body weight. Also, lesion size and spleen parasite burden decreased in comparison to no treatment group.

**Conclusion:**

The alcoholic extract could be a new alternative treatment for cutaneous leishmaniasis. However this extract needs more investigation for novel herbal drugs against CL.

## Introduction

Leishmaniasis is considered one of the six major neglected tropical diseases (NTDs) by the World Health Organization (WHO) (1). Currently, 13 million people in 98 countries have different types of leishmaniasis (2), and according to a 2017 report from the World Health Organization, about 600,000 to 1 million new cases each year have become a public health hazard worldwide (https://www.who.int/newsroom/fact-sheets/detail/leishmaniasis). The most common form of leishmaniasis is the cutaneous form caused by the protozoan parasites belonging to the genus *Leishmania* (3,4). Drug resistance, limitary efficacy and severe side effects of common drugs such as sodium stibogluconate and meglumine antimoniate remain a challenge for treatment of cutaneous leishmaniasis (5). Therefore, these agents provide the need for a new effective antileishmanial drug.

Natural products and their compounds have long been used in the production of new drugs because they are cost-effective and easily accessible (6). Sweet fennel (*Foeniculum vulgare*) is known as a medicinal and aromatic plant belonging to the *Apiaceae* (7,8). Anti-microbial properties of *Foeniculum vulgare* extract have been confirmed on bacteria and fungi (9,10) so that it may affect the *Leishmania* parasite.

This is the first study that has been done on the anti-parasitic characteristics of *F. vulgare* aqueous and alcoholic seed extract against cutaneous leishmaniasis (CL) due to *L. major*. In *in vivo,* the most effective fennel extract, its cytotoxicity effects on promastigotes, macrophages and infected macrophages have been assessed. Lesion size and parasite burden of the spleen were measured in the infected BALB/c mice.

## Materials and Methods

**Preparation of plant extract**: The *F. vulgare* seeds were obtained from the Barij Essence Pharmaceutical Company (Iran). Plant seeds were ground and macerated. Twenty-five grams of powder was solvated with 200 ml methanol or distilled water and was shaken for 72 hours at 25°C. After filtration through whatman filter paper (No. 4), re-extraction was carried out two times, and then the extracts of every sample were evaporated (25°C) and dehydrated in desiccators under vacuum to a constant weight. At the end, products were sterilized entirely by filtration with a 0.22 µm membrane filter. Finally, the alcoholic and aqueous extracts were applied in the project freshly.

**Macrophage and parasite cultures**: BALB/c mice-derived macrophage cell line RAW 264.7 (ATCC® TIB-71™) (Iranian Biological Resource Center, Tehran, Iran) was cultured in High-glucose Dulbecco's Modified Eagle Medium (DMEM) (Bioidea, Iran) with 10% Heat-inactivated Fetal Bovine Serum (FBS) (Gibco, USA) and 1% Penicillin-streptomycin (pen/strep) (10,000 U/mL) (Gibco, USA). RAW 264.7 cells kept at 37°C in 5% CO2 (pH 7.6). The passage of the RAW 264.7 cell line was accomplished by cell scraper after every 3 days. *L. major* promastigotes (MHROM/IR/75/ER) (Department of Parasitology, Tehran University of Medical Sciences, Tehran, Iran) cultured at 25 °C in RPMI 1640 (Biosera, France) supplemented with 10% FBS and 1% pen/strep. The passage of *Leishmania* parasite was done almost after 5 days according to medium color.

***In-vitro* cytotoxicity assay of the alcoholic and aqueous extracts on macrophages (CC50)**: In order to determine the cytotoxicity dosage of *F. vulgare* extract, the RAW 264.7 cells (1×10^5^ cells) were seeded in the presence of the extract growing concentrations (10 to 4500 µg/ml) in 96-well microliter culture plates in 5% CO2 for 48 h at 37 °C. Cell viabilities were measured using colorimetric 3-(4,5 dimethylthiazol-2-yl)-2,5-diphenyl tetrazolium bromide (MTT) (Sigma-Aldrich, Deisenhofen, Germany) assay as it was described earlier (11). No treatment and glucantime® (5 mg/mL) were used as controls. Absorbance ratios were measured at 530 nm wavelength. These results were indicated as the mean percentage (%) reduction of macrophages in comparison with untreated control samples × 100. Finally, the concentration producing 50% (µg/ml) (CC50) was determined with respect to the method performed by Weislow et al. (12). CC50 amounts were obtained using Prism 8 software (Graph-Pad Prism, San Diego, California, USA).

***In-vitro* cytotoxicity assay of the extracts on promastigotes (IC50)**: The effects of rising concentrations of *F. vulgare* extract (10 to 4500 µg/ml) on the stationary phase of *L. major* promastigotes (1×10^6^ parasites) were quantified for 48 hours at 26 °C. The susceptibility was determined by counting with Neubauer chamber (13) and the extract concentration was estimated, which resulted in 50% inhibition in promastigotes growth (µg/ml) (IC50).

***In-vitro* cytotoxicity assay of the extracts on infected macrophages by *Leishmania* (EC50)**: RAW 264.7 macrophages (2×10^6^ cells) were cultured onto a crystal chamber slide, along with in 24-well culture plate in DMEM medium supplemented with 10% FBS, which was incubated for 24 hours in 5% CO2 at 37°C. *L. major* promastigotes were cultured at 25°C in RPMI-1640 with 10% FBS to reach stationary growth phase. Then, to infect RAW 264.7 macrophages, promastigotes were added to each chamber slide and 24-wells at the ratio of 1:10 with parasite- to-host, followed by incubation for 24 hours. Free promastigotes were removed by washing two times using serum-free RPMI-1640 medium. Finally, infected macrophages were treated with increasing concentrations (10 to 4500 µg/ml) of *F. vulgare* extract for 48 hours at 37 °C in 5% CO2 as it was described earlier. After 48 hours, RPMI 1640 medium was removed and 50 µL PBS was added. Then, it was scraped by scraper and transfered to slide. At the end of this stage, the slide was stained with Giemsa. The antileishmanial effect of the *F. vulgare* extract was assessed by microscopic method. Hundred macrophages were counted, and infected macrophage was reported as percent.

**Selectivity Index (SI) Determination**: Herein, the ratio of CC50 value of the cytotoxic activity to EC50 value of the antileishmanial activity was determined; then, the extract SI was calculated (14). Furthermore, SI of promastigotes was calculated (SI = CC50 Macrophages/IC50 promastigote) (15). SI value under 10 shows ideal antileishmanial activity for this compound. Briefly, the ideal herbal compounds have the low cytotoxic, and they have the high antileishmanial activity (higher reported values = greater extract activity) (14).

**Ethical statements**: All animal experimental procedures of this study were ratified by the Human and Animal Research Ethics Committee of Aja University of Medical Sciences (ethical code: IR. AJAUMS.MSP.REC1398.151). This study was done with respect to the guidelines of the Specific National Ethics for Biochemical Research issued by the Research and Technology Deputy of the Ministry of Health of Iran (issued in 2005).

**Mice**: All efforts were accomplished to decrease BALB/c mice suffering throughout the experiment period. All the mice were female of 5±1 week's old or 20g weight that were purchased from Iran Pasteur Institute. Mice were placed in plastic cages and with free access to enough food and drinking water. BALB/c mice were kept in a controlled animal care facility including 21–25°C, humidity 57±2%, and 12 hours of light-dark cycles.

**Parasite strain and mice infection**: The *Leishmania* parasite was sustained in a high virulent state throughout a continuous passage in susceptible BALB/c mice. The spleen tissues from infected BALB/c mice were squished and cultured at 26°C in RPMI 1640 medium supplemented with 10% FBS containing 1% pen/strep. All 20 laboratory mice were inoculated subcutaneously (SC) into the tail base, with *Leishmania* promastigotes (2×10^6^) at liquid stationary phase culture. After thirty days, nodules and lesions emerged on 20 inoculated mice.

**Treatment of infected mice with *F. vulgare* alcoholic extract**: To test the *F. vulgare* extract effects on infected BALB/c mice, all mice were distributed into four separate groups: A, B, C, D, (five mice per each group). Group A was control group (received no treatment); group B received *F. vulgare* intralesionally (IL) according to *in vitro* (300µL; 4500 µg/mL); group C received glucantime® intralesionally (IL) (injections of 20 mg/kg); group D received PBS as placebo (three times per week for 4 weeks).

**Measurement of the lesion size and mice body weight**: Size of the lesions and mice body weight was measured before and after 4 weeks of treatment using a metric caliper (Mitutoyo, Taiwan) and lab balance (Ohaus, Switzerland), respectively.

**Quantification of *Leishmania* parasite load in lesion region**: To measure the parasite load of the lesions, sampling was performed from the margins of lesions by using a vaccinostyle, and then smears were prepared. Smears on glass slides were fixed with absolute methanol, stained with Giemsa. Slides were evaluated for the presence of amastigotes using Zeiss light microscope (Carl Zeiss, Germany).

**Quantification of *Leishmania* parasite load using limiting dilution assay (LDA)**: After eight weeks from infection, the *Leishmania* parasite load in the spleen was quantified by LDA. Furthermore, five mice from each group were scarified. Then, spleen was ground by 100 µm cell strainer and was transferred into the culture media;, amastigotes were transformed to the motile active promastigotes. Finally, motile promastigotes were counted by invert microscopy and parasite load was assessed throughout, limiting dilution assay test as it was described earlier (16).

**Statistical analysis**: Statistical analyses of all cytotoxicity assays including CC50, EC50, and IC50 were performed by the use of Prism 8.0 software. Both one-way ANOVA and Student's t-test were used. Furthermore, statistical differences were supposed significant at *p*-values less than 0.05. It is worth mentioning that all tests of our study were accomplished in duplicate.

## Results

**Figure 1:**
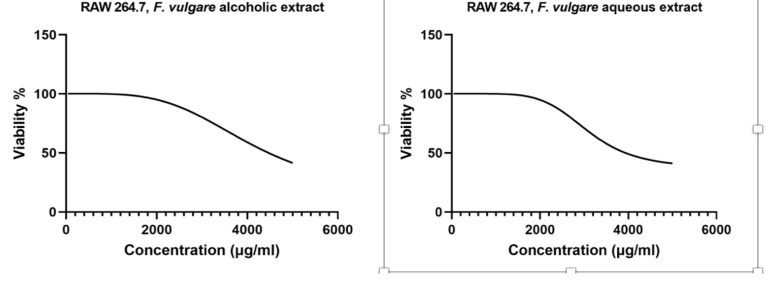
Cytotoxicity assay (CC50) of concentrations of F. vulgare alcoholic and aqueous extracts on mouse RAW 264.7 macrophage cell line after 48 hours using MTT method in vitro

**Cytotoxicity effects of *F. vulgare* extracts on macrophages (CC50)**: As shown in Figure 1, *F. vulgare* extract was only toxic for RAW 264.7 macrophages at high extract concentrations. The CC50 (µg/ml) values of *F. vulgare* alcoholic and aqueous extracts at 48 hours were 4500 µg/ml and 3900 µg/ml, respectively.

**Figure 2:**
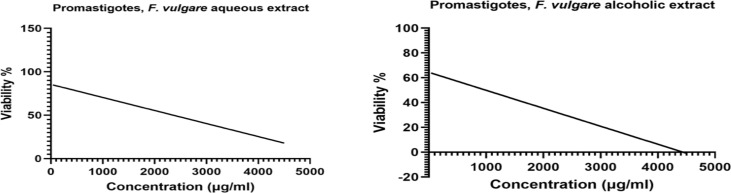
An inhibitory assay (IC50) of F. vulgare alcoholic and aqueous extracts concentrations on L. major promastigotes after 48 hours by using Neubauer chamber counting

**Antileishmanial effects of *F. vulgare* extracts on *Leishmania* promastigotes (IC50)**: The *F. vulgare* alcoholic and aqueous extracts strongly inhibited the *Leishmania* parasite growth with the IC50 of 1200µg/ml and 2500 µg/ml, respectively at 48 hours (Figure 2).

**Figure 3:**
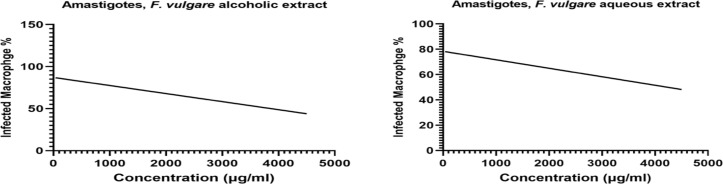
An effective concentration (EC50) of F. vulgare alcoholic and aqueous extracts L. major on L. major promastigotes after 48 hours by using light microscopy method

**Parasite Rescue and Transformation Assay (PRTA or transformed promastigotes) (EC50)**: As indicated in Figure 3, the *F. vulgare* alcoholic and aqueous extracts could inhibit the growth of *Leishmania* amastigotes, EC50 3900 µg/ml and EC50 4250 µg/ml, respectively (Fig 3). Therefore, *F. vulgare* extract did not indicate any negative/toxic effects on mice RAW 264.7 macrophage cell line, but it could inhibit the intracellular amastigotes growth and kill the parasite (Figures 1 and 3).

***F. vulgare* extracts and selectivity index (SI)**: *F. vulgare* alcoholic and aqueous extracts were active against *L. major* amastigotes (1.15 µg/ml and 0.9 µg/ml, respectively) and promastigotes (3.75 µg/ml and 1.56 µg/ml, respectively) of *L. major* with a favorable SI compared to RAW 264.7 cell line macrophages. SI results were shown that alcoholic extract was more effective than aqueous extract.

**Lesion size in infected BALB/c mice treatment with *F. vulgare* alcoholic extract**: To measure the effect of *F. vulgare* alcoholic extract on the experimental CL development, BALB/c mice were infected with stationary phase *L. major*. Afterwards, the development of CL lesion was monitored 8 weeks. Although CL lesions size in group B had not significant variation before and after treatment with *F. vulgare* alcoholic extract, it was reduced significantly as compared with A and D groups (Table 1) (*p*<0.05). Even if body weight of BALB/c mice in treated group with *F. vulgare* alcoholic extract (B group) had not considerable differences with prior treatment, body weight of this group increased significantly in comparison with the control group(A group) (*p*<0.05) (Table 2).

**Table 1:** Variation of lesion size (mm^2^) of infected BALB/c mice, before and after treatment

Groups	Before treatment	After 4 weeks treatment	*p* value (before/after)	*p* value (compare to negative control)
***F. vulgare* alcoholic extract**	13.4±3.8	20 ± 5.4	0.065	<0.0001
**PBS (vehicle)**	14.3±3.7	37.3±8.9	<0.0001	0.081
**Glucantime®**	12.4±2.3	9.1± 2.1	0.356	<0.0001
**Negative Control**	15.1±2.8	43.6± 9.2	<0.0001	-----

Values are represented as mean ± SD, Positive control (Glucantime®): the mice were treated twice a week till four weeks with 300 µl (5 mg/ml) intralesional injection. (*p*<0.05)

**Table 2:** . Variation of body weight (gr) of infected BALB/c mice, before and after treatment

Groups	Before treatment	After 4 weeks treatment	*p* value (before/after)	*p* value (compare to negative control)
***F. vulgare* alcoholic extract**	17.0±1.0	17.1±1.0	0.950	0.049
**PBS (vehicle)**	16.9±1.3	16.3±0.7	0.368	0.082
**Glucantime®**	15.9±0.8	15.4±0.4	0.513	0.827
**Negative Control**	16.8±1.3	15.7±0.7	0.111	------

Values are represented as mean ± SD, Positive control (Glucantime®): the mice were treated twice a week till four weeks with 300 µl (5 mg/ml) intralesional injection (*p*<0.05)

**Fig 4:**
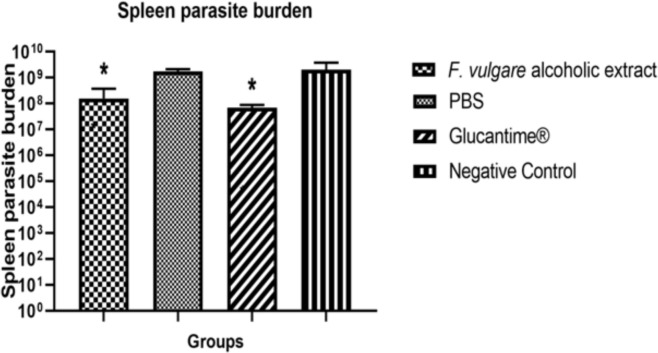
Spleen parasite burden in infected BALB/c mice after 4 weeks by using LDA (*p*<0.05).

**Parasite burden in lesion and spleen of infected BALB/c mice**: *F. vulgare* alcoholic extract reduced significantly parasite burden in BALB/c mice lesions in comparison to no treatment group (*p*<0.05). Four weeks after treatment, the *L. major* burden in the draining spleen of BALB/c mice was evaluated using limiting dilution assay (LDA). The *L. major* parasite burden was significantly reduced in *F. vulgare* alcoholic extract group compared to no treatment and PBS groups (Figure 4) (*p*<0.05).

## Discussion

Traditionally, attempts have been made to use herbal plants to treat cutaneous leishmaniasis (17). As previously mentioned, resistance to chemical drugs and their side effects have been reported in the treatment of cutaneous leishmaniasis (5). Herbal useful remedies could be used to replace chemical treatments for cutaneous leishmaniasis treatment (18). *F. vulgare,* belonging to the *Umbelliferae* (*Apiaceae*) family, is a plant native to the Mediterranean area. Fennel are cultivated in different regions of Europe and Asia, and most of them are imported from India, China and Egypt (19–21). Various studies have reported on the effect of this plant extract on genital system, respiratory disorders and digestive system (22,23). *F. vulgare* is known as anti-microbial, anti-inflammatory, anti-diabetic, anti-tumor, with low toxicity and has immunomodulatory effect (9,22,24,25). This study showed the therapeutic effects of aqueous and alcoholic extracts of *F. vulgare* as herbal drug without extreme toxicity to host cells. Determination of anti-leishmanial activity of *F. vulgare* extracts were accomplished with IC50, CC50 and EC50. The results of this research revealed that the optimal concentrations of the alcoholic extract for reducing the promastigotes and amastigotes growth were 1200 and 3900 µg/ml, respectively. According to this study, *F. vulgare* alcoholic extract has a potent action against *L. major* promastigotes and amastigotes whereas it doesn't have any harmful side effect on the host macrophages. Two main components including bis (2-ethylhexyl) phthalate and trans-anethole, are predominantly responsible for most of antileishmanial effects (26). Kataoka et al. showed that the fennel extract could inhibit inflammatory event (27). The suppressive effects of methanol extract against acute and subacute sickness, type 4 allergic reactions through cyclooxygenase and lipoxygenase inhibition have already been confirmed (28). There are active compounds with anti-microbial activity such as oleic acid and coumarin in aqueous and alcoholic extracts (29,30). The prenylated coumarins auraptene, galbanic acid and umbelliprenin isolated from another genus *Apiaceae* (*Ferula szowitsiana*) were revealed *invitro* antileishmanial effects on *L. major* promastigotes in comparison with the negative control (31, 32, 33). The SI was known as a major index of herbal extract activity that is used in order to describe a compound's *in vitro* efficacy in the parasite proliferation inhibition (15). This index revealed that the *F. vulgare* alcoholic extract was more toxic to *L. major* parasite in comparison with the macrophages. Indeed, the alcoholic extract indicated a remarkable anti-leishmanial activity, and has the potency to decrease the survival rate of amastigotes at nontoxic concentrations for the host cell. After treatment of infected BALB/c mice with *F. vulgare* alcoholic extract, all of the treatment groups did not show any increase in lesions size in comparison with no treatment and PBS groups. The LDA and light microscopy method demonstrated a considerable difference between the parasite burden in the *F. vulgare* extract and a glucantime® treated groups, in comparison with PBS and no treatment. Also, a significant increase in body weight of *F. vulgare* alcoholic extract treated mice showed that this extract had no toxic side effects on mice growth. In this study, methanolic extract is more effective than aqueous extract to killing *Leishmania* parasite but toxic for mammalian cells. As shown in Hamdy Roby and *et al* study, using other solvent such as hexane and diethyl ether can be found having better result against *Leishmania major* (10). Many studies have been conducted on effective drugs in order to decrease infection by parasites. Some research has been done on the use of herbal drugs on *L. major* parasites. Various types of drug delivery and nanoparticle therapy were examined in order to determine the effectiveness of herbal materials and disease treatments by pharmaceutical companies. In Iran, different types of herbal extracts have been considered for treating the *L. major* infection. Previously, no evidences reported on the anti-leishmanial effects of *F. vulgare* alcoholic extract in a mice model. This herbal extract could be a new alternative treatment for cutaneous leishmaniasis. Therefore, *F. vulgare* alcoholic extract with more investigation could be a candidate for novel herbal drugs against neglected tropical diseases such as leishmaniasis, and its promising effects for future studies is considerable.
